# Nonconsensus Protein Binding to Repetitive DNA Sequence Elements Significantly Affects Eukaryotic Genomes

**DOI:** 10.1371/journal.pcbi.1004429

**Published:** 2015-08-18

**Authors:** Ariel Afek, Hila Cohen, Shiran Barber-Zucker, Raluca Gordân, David B. Lukatsky

**Affiliations:** 1 Department of Chemistry, Ben-Gurion University of the Negev, Beer-Sheva, Israel; 2 Center for Genomic and Computational Biology, Department of Biostatistics and Bioinformatics, Duke University, Durham, North Carolina, United States of America; Ottawa University, CANADA

## Abstract

Recent genome-wide experiments in different eukaryotic genomes provide an unprecedented view of transcription factor (TF) binding locations and of nucleosome occupancy. These experiments revealed that a large fraction of TF binding events occur in regions where only a small number of specific TF binding sites (TFBSs) have been detected. Furthermore, *in vitro* protein-DNA binding measurements performed for hundreds of TFs indicate that TFs are bound with wide range of affinities to different DNA sequences that lack known consensus motifs. These observations have thus challenged the classical picture of specific protein-DNA binding and strongly suggest the existence of additional recognition mechanisms that affect protein-DNA binding preferences. We have previously demonstrated that repetitive DNA sequence elements characterized by certain symmetries statistically affect protein-DNA binding preferences. We call this binding mechanism *nonconsensus protein-DNA binding* in order to emphasize the point that specific consensus TFBSs do not contribute to this effect. In this paper, using the simple statistical mechanics model developed previously, we calculate the nonconsensus protein-DNA binding free energy for the entire *C*. *elegans* and *D*. *melanogaster* genomes. Using the available chromatin immunoprecipitation followed by sequencing (ChIP-seq) results on TF-DNA binding preferences for ~100 TFs, we show that DNA sequences characterized by low predicted free energy of nonconsensus binding have statistically higher experimental TF occupancy and lower nucleosome occupancy than sequences characterized by high free energy of nonconsensus binding. This is in agreement with our previous analysis performed for the yeast genome. We suggest therefore that nonconsensus protein-DNA binding assists the formation of nucleosome-free regions, as TFs outcompete nucleosomes at genomic locations with enhanced nonconsensus binding. In addition, here we perform a new, large-scale analysis using *in vitro* TF-DNA preferences obtained from the universal protein binding microarrays (PBM) for ~90 eukaryotic TFs belonging to 22 different DNA-binding domain types. As a result of this new analysis, we conclude that nonconsensus protein-DNA binding is a widespread phenomenon that significantly affects protein-DNA binding preferences and need not require the presence of consensus (specific) TFBSs in order to achieve genome-wide TF-DNA binding specificity.

## Author Summary

Interactions between proteins and DNA trigger many important biological processes. Therefore, to fully understand how the information encoded on the DNA transcribes into RNA, which in turn translates into proteins in the cell, we need to unravel the molecular design principles of protein-DNA interactions. It is known that many interactions occur when a protein is attracted to a specific short segment on the DNA called a specific protein-DNA binding motif. Strikingly, recent experiments revealed that many regulatory proteins reproducibly bind to different regions on the DNA lacking such specific motifs. This suggests that fundamental molecular mechanisms responsible for protein-DNA recognition specificity are not fully understood. Here, using high-throughput protein-DNA binding data obtained by two entirely different methods for ~100 TFs in each case, we show that DNA regions possessing certain repetitive sequence elements exert the statistical attractive potential on DNA-binding proteins, and as a result, such DNA regions are enriched in bound proteins. This is in agreement with our previous analysis performed for the yeast genome. We use the term *nonconsensus protein-DNA binding* in order to describe protein-DNA interactions that occur in the absence of specific protein-DNA binding motifs. Here we demonstrate that the identified nonconsensus effect is highly significant for a variety of organismal genomes and it affects protein-DNA binding preferences and nucleosome occupancy at the genome-wide level.

## Introduction

Binding of TFs to their target sites on the DNA is a key step during gene activation and repression. An existing paradigm assumes that the main mechanism responsible for specific TF-DNA recognition is TF binding to short (typically 6–20 bp long) DNA sequences called *specific consensus motifs*, or *specific TF binding sites* (TFBSs). It has been known for a long time, since the seminal studies of Iyer and Struhl [1], that genomic context surrounding specific TFBSs significantly influences TF-DNA binding preferences. However, general rules describing the mechanisms responsible for such influences remain unknown.

Recently, the model organism ENCODE (modENCODE) project has revealed genome-wide comprehensive maps of TF-DNA binding and nucleosome occupancy in *C*. *elegans* [2–7] and in *D*. *melanogaster* [8–10]. Remarkably, these studies have challenged the existing paradigm and revealed that a large fraction of TF-DNA binding events occurs in genomic regions depleted of specific consensus motifs. Such genomic regions with enhanced overall TF-DNA binding but depleted in consensus motifs are oftentimes of low sequence complexity, which means that they are enriched in repeated DNA sequences.

We have recently proposed that repetitive DNA sequences characterized by certain symmetries and length scales of repetitive sequence patterns (see below) exert a statistical potential on DNA-binding proteins, affecting their binding preferences [11–15]. This effect of protein binding to repetitive DNA sequences in the absence of specific base-pair recognition is different from the concept of nonspecific protein-DNA binding introduced and explored in seminal studies of von Hippel, Berg, et al. [16–21]. In particular, von Hippel and Berg defined two related mechanisms for nonspecific protein-DNA binding [19]. The first mechanism is DNA sequence-independent, and it assumes that DNA exerts an electrostatic attraction upon DNA-binding proteins, modulated by the overall DNA geometry [19]. It has been proposed that DNA-binding proteins use different conformations in specific and nonspecific binding modes [16–20, 22]. The second mechanism assumes that mutated specific DNA consensus motifs retain a reduced binding affinity for sequence-specific TFs [19]. Nonspecific protein-DNA binding might become significant since the statistical probability to find such imperfect motifs in many genomic locations by random chance is high for eukaryotic genomes [19, 23]. The importance of nonspecific protein-DNA binding has been experimentally demonstrated for a number of systems both *in vivo* [24, 25] and *in vitro* [26–31].

We demonstrated recently that repetitive DNA sequence patterns characterized by certain symmetries lead to *nonconsensus protein-DNA binding* that can be enhanced or reduced depending on the symmetry type [11]. We use the term *nonconsensus protein-DNA binding* in order to emphasize the point that the nonconsensus protein-DNA binding free energy is computed without using any experimental information on specific protein-DNA binding preferences (see below). For example, we showed that repetitive homo-oligonucleotide sequence patterns, such as repeated poly(A)/poly(T)/poly(C)/poly(G) tracts lead to statistically enhanced nonconsensus protein-DNA binding affinity [11]. Our results indicated that such nonconsensus binding significantly influences nucleosome occupancy [12], TF-DNA binding preferences [13], and transcription pre-initiation complex binding preferences [14] in yeast.

In addition, using the protein binding microarray (PBM) method, we have recently directly measured the nonconsensus protein-DNA binding free energy for several human TFs [15]. We have demonstrated that, remarkably, the magnitude of the identified nonconsensus effect reaches as much as 66% of consensus (specific) binding [15].

In this study we explore the extent and significance of the nonconsensus protein-DNA binding mechanism for a large number of proteins belonging to different structural families. First, we investigate the nonconsensus effect in more complex, multicellular organisms, using the available ChIP-seq data obtained for ~100 TFs in *C*. *elegans* [2, 3] and *D*. *melanogaster* [10, 32]. Next, we perform the analysis of high-resolution *in vitro* universal protein-DNA binding microarray (PBM) data obtained for ~90 eukaryotic TFs belonging to 22 different DNA-binding domain types [33–35]. In addition, we identify protein sequence features that statistically distinguish between proteins with stronger and weaker response to nonconsensus repetitive DNA sequence elements, respectively.

We stress the point that *in vitro* analysis is free of confounding factors present in a cell, such as nucleosomes and indirect TF-DNA binding. Our previous experimental *in vitro* study of nonconsensus protein-DNA binding was performed for only 6 TFs [15]. The present analysis of the vast amount of *in vitro* TF-DNA binding data extends this number to more than an order of magnitude, suggesting that the nonconsensus mechanism most likely represents the statistical law rather than the exception. Therefore, the results reported here strongly support our conclusion that nonconsensus protein-DNA binding is a widespread phenomenon that significantly affects protein-DNA binding preferences in eukaryotic genomes, and need not require the presence of consensus (specific) TFBSs in order to achieve genome-wide TF-DNA binding specificity.

## Results

### Nonconsensus free energy correlates with *C*. *elegans* and *D*. *melanogaster* TF-DNA binding preferences

**Fig 1:**
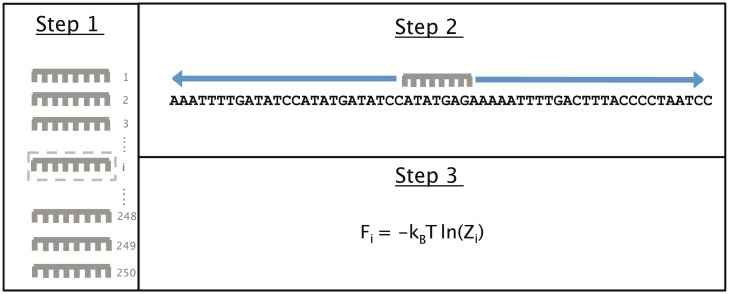
Cartoon illustrating our model for computing the free energy of nonconsensus protein-DNA binding. Schematic representation of the procedure for computing the nonconsensus free energy. Step 1: In order to model nonspecific TF-DNA binding, we generate an ensemble of 250 *random* TFs. Step 2: Each TF moves within a sliding window of width *L* bp. The TF-DNA binding energy is computed at each location of TF along the sliding window using the random potential. Step 3: For each TF we calculate the TF-DNA binding free energy. We repeat this process for all random TFs and compute the *average* nonconsensus binding free energy with respect to this ensemble of random TFs. Moving the sliding window along the genome, we assign the nonconsensus TF-DNA binding free energy at each genomic location. We assume that each random binder makes contacts with *M* bps upon DNA binding. For each model TF (random binder), we define the partition function of protein-DNA binding within the chosen sliding window of width *L* bp. We used *L* = 50 bp (i.e. the sliding window size) in our calculations of the genome-wide nonconsensus TF-DNA binding free energy profiles, and *L* = 36 bp for calculations of the nonconsensus TF-DNA free energies for *in vitro* protein binding microarray (PBM) experiments. In the latter case, we do not move the sliding window since each DNA sequence in the PBM library is 36-bp long.

We compared the predicted landscape of *nonconsensus protein-DNA binding free energy* with the genomic binding profiles of 69 transcriptional regulators in *C*. *elegans* [2, 3] and 30 transcriptional regulators in *D*. *melanogaster* [10, 32], as determined by ChIP-seq in the modENCODE project [2, 3, 8, 10]. We computed the nonconsensus binding free energy landscape using a simple approach that we developed previously [11]. Briefly, we used a set of random protein-DNA binders as a proxy for nonspecific protein-DNA interactions in a crowded cellular environment (Fig 1). Next, to each location along the *C*. *elegans* and *D*. *melanogaster* genomes, we assigned an average *free energy of nonconsensus protein-DNA binding*, 〈*F*〉_*TF*_, where the averaging is performed over an ensemble of random binders (see Methods for further details). The free energy value at each sequence location is entropy-dominated, and it is influenced exclusively by the presence of repetitive DNA sequence patterns [11] surrounding that location. We use the term *DNA sequence correlations* to describe the repetitive DNA patterns, and the term *correlation scale* to describe the length of the patterns (Methods). The larger the correlation scale, the larger the number of repetitive sequence patterns, and thus the stronger the nonconsensus protein-DNA binding effect [11]. Importantly, the genomic DNA sequence constitutes the only input for the nonconsensus binding model, i.e. the model does not have any fitting parameters (Methods).

**Fig 2:**
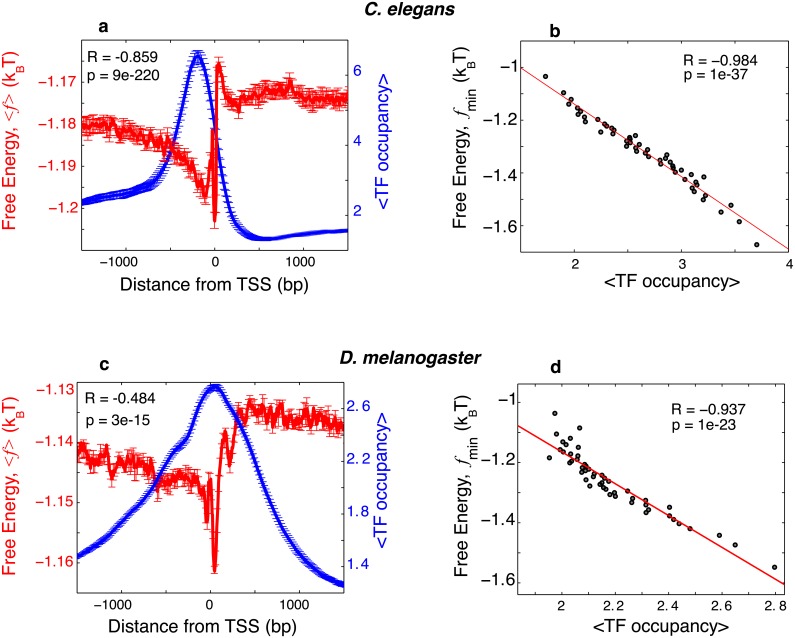
The free energy of nonconsensus TF-DNA binding negatively correlates with the combined TF occupancy for both *C*. *elegans* and *D*. *melanogaster* genomes. (**a**) The average *free energy of nonconsensus TF-DNA binding* per bp, $\langle f\rangle ={\langle {\langle F\rangle }_{TF}\rangle }_{seq}/M$ (red), and the average, combined occupancy profile of 69 *C*. *elegans* TFs (blue), plotted around the TSSs of 17,207 *C*. *elegans* coding genes. The notation <TF occupancy> describes the average, combined occupancy profile of all 69 TFs. The linear correlation coefficient is computed for a linear fit of 〈*f*〉 versus <TF occupancy> at individual genomic locations, computed every 4 bp, within the interval (-1500,1500) around the TSS. The sequences are aligned with respect to the TSS. In order to compute error bars, we divided genes into ten randomly chosen subgroups, and computed 〈*f*〉 for each subgroup. The error bars are defined as one standard deviation of 〈*f*〉 between the subgroups. The error bars for the combined TF occupancy are computed analogously. (**b**) Correlation between the minimum value of the free energy of nonconsensus TF-DNA binding, *f*_min_ = min(*f*), and the combined occupancy of all TFs, computed for individual genes in non-overlapping windows of 100 bp, within the entire interval (-1000,1000). The data was grouped into 50 bins. (**c**) Similar to (a) but showing the average free energy of nonconsensus TF-DNA binding per bp, 〈*f*〉 (red), and the average transcription factor occupancy (blue), around the TSSs of 12,188 *D*. *melanogaster* genes. (**d**) Similar to (b) but for 12,188 *D*. *melanogaster* genes.

We found that the nonconsensus protein-DNA binding free energy correlates negatively with the combined TF occupancy in both the *C*. *elegans* and the *D*. *melanogaster* genomes, i.e. the lower the nonconsensus binding free energy, the higher the combined TF occupancy (Fig 2). Fig 2a and 2c illustrate this correlation for free energy profiles, ${\langle {\langle F\rangle }_{TF}\rangle }_{seq}$, averaged over genomic sequences aligned with respect to the TSS. A statistically significant correlation at the single gene level is also observed, on average, without sequence alignment with respect to the TSS (Fig 2b and 2d). In these analyses both genomes show statistically significant negative correlations, with the correlation being more pronounced in *C*. *elegans*.

We verified that the predicted free energy landscape is qualitatively robust with respect to variations in the model parameters (i.e. the sliding window width, *L*, and the TFBS size, *M*) (S1 Fig). In addition, we validated that the predicted free energy landscape is determined by the presence of repetitive sequence patterns, and *not* by the average genomic nucleotide content. To show this, we shuffled the DNA sequence in each sliding window along the genome to obtain random DNA sequences with a fixed nucleotide content, and we computed the normalized free energy, *δF* = *F*−*F*_rand_, where *F*_*rand*_ is the free energy of the random, shuffled sequences, averaged over different random realizations (Methods). As shown in S2 Fig, the normalized free energy *δF* is robust with respect to global variations in the genomic nucleotide content.

The predicted reduction in the nonconsensus free energy upstream of TSSs (Fig 2a and 2c) stems from the enhanced level of homo-oligonucleotide sequence correlations (i.e. repetitive homo-oligonucleotide sequence patterns, such as repeated poly(dA:dT) tracts). This effect can be intuitively understood in the following way. As shown in our previous work, the presence of enhanced homo-oligonucleotide sequence correlations within a DNA region generally leads to the widening of the protein-DNA binding energy spectrum in this region [11]. For example, in the statistical ensemble of random binders interacting with DNA sequence that contains long homo-oligonucleotide tracts with two alternating types of nucleotides (such as alternating poly(dA:dT) and poly(dT:dA) tracts), the width (i.e. the standard deviation) of the binding energy spectrum, $\sigma_{U}^{\text{homo}}$, will be universally larger than the corresponding width for the case of entirely random DNA sequence, $\sigma_{U}^{\text{homo}}≃\sqrt{2}\cdot \sigma_{U}^{\text{random}}$ [11]. This result is independent of the microscopic details of the protein-DNA interaction potential, *U*, and it is simply the consequence of the central limit theorem [36, 37]. The wider energy spectrum, $\sigma_{U}^{\text{homo}}>\sigma_{U}^{\text{random}}$, universally leads to the statistically lower free energy, *F* ^homo^ *< F* ^random^ [38], and therefore to a higher nonconsensus protein-DNA binding affinity. The computed probability distributions of the nonconsensus protein-DNA binding energy and the free energy in the *C*. *elegans* genome, further illustrates this mechanism (S3 Fig). Thus, the nonconsensus protein-DNA binding mechanism can significantly influence TF-DNA binding preferences in the *C*. *elegans* and *D*. *melanogaster* genomes, complementing the conventional, specific protein-DNA recognition mode.

**Fig 3:**
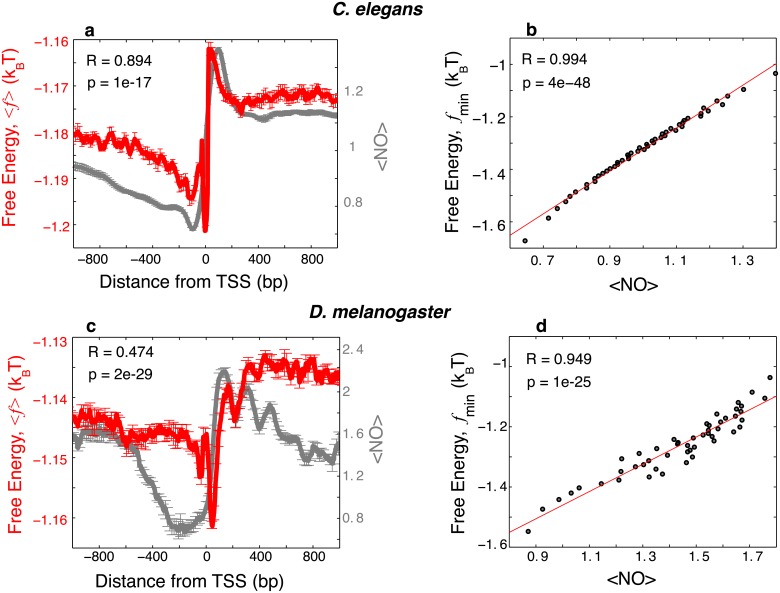
The free energy of nonconsensus TF-DNA binding positively correlates with the nucleosome occupancy. (**a**) The average free energy of nonconsensus TF-DNA binding per bp, $\langle f\rangle ={\langle {\langle F\rangle }_{TF}\rangle }_{seq}/M$ (red), and the average nucleosome occupancy from [4] (gray), around the TSSs of 23,287 mRNA coding and non-coding *C*. *elegans* genes. The linear correlation coefficient is computed for a linear fit of 〈*f*〉 versus the average nucleosome occupancy at individual genomic locations, computed every 4 bp, within the interval (-1000,1000). In order to compute error bars, we divided genes into five randomly chosen subgroups, and computed 〈*f*〉 for each subgroup. The error bars are defined as one standard deviation of 〈*f*〉 between the subgroups. (**b**) Correlation between the minimal value of the free energy of nonconsensus TF-DNA binding, *f*_min_ = min(*f*), and the nucleosome occupancy, computed for individual genes in non-overlapping windows of 100 bp within the interval (-1000,1000) around the TSS for each of the 23,287 genes. The data was grouped into 50 bins. **(c**) Similar to (a) but showing the average free energy of nonconsensus TF-DNA binding per bp, 〈*f*〉 (red), and the average H2A.Z nucleosome occupancy (grey) around the TSSs of 12,188 *D*. *melanogaster* genes [32]. (**d**) Similar to (b) but for 12,188 *D*. *melanogaster* genes.

We stress the fact that the minimum of the *average* nonconsensus protein-DNA binding free energy landscape does not align precisely with the maximum of the average TF occupancy profile in both *C*. *Elegans* and *D*. *melanogaster* genomes (Fig 2a and 2c). Such mismatch is also observed between the average nonconsensus protein-DNA binding free energy landscape and the average nucleosome profile (see below, Fig 3a and 3c), similar to the case as we previously observed for the yeast genome [12]. Combination of additional factors not taken into account in our model but present *in vivo* might explain a possible origin of such a mismatch. These factors include, first, steric constrains imposed by the presence of nucleosome particles [39]; second, steric constrains imposed by the transcription pre-initiation complex (PIC) [40]; and third, the presence of specific TFBSs [41].

### Nonconsensus protein-DNA binding influences nucleosome preferences

We also assessed the effect of nonconsensus protein-DNA binding on nucleosome binding preferences in the *C*. *elegans* and *D*. *melanogaster* genomes. Genome-wide measurements of nucleosome occupancy show a typical nucleosome depleted region upstream of the TSSs, and a well-positioned +1 nucleosome [2, 4, 42]. In *D*. *melanogaster*, an oscillating nucleosome occupancy pattern was observed, similar to the one in yeast [43], while the *C*. *elegans* genome-wide nucleosome occupancy profile does not demonstrate such strong oscillations [4, 42].

The computed nonconsensus free energy landscapes show a statistically high, positive correlation with the nucleosome occupancy profile in both genomes (Fig 3). In particular, the average nonconsensus free energy shows a pronounced minimum in the upstream nucleosome depleted region (Fig 3a and 3c), similar to the one observed in yeast [12]. In Fig 3b and 3d we also observed, at the single gene level, statistically significant correlation between the average nucleosome occupancy and the average free energy of nonconsensus binding (Methods). Sequences with lower nonconsensus protein-DNA binding free energy have, on average, lower nucleosome occupancy.

We suggest that the observed effect stems from the competition between TFs that experience enhanced nonspecific attraction towards upstream promoter regions (i.e., reduced level of the nonconsensus free energy) and nucleosome-forming histones. It is important to stress that the presence of repetitive DNA sequence elements in promoter regions might also affect histone-DNA binding due to the nonconsensus mechanism, and as a result of it, the nucleosome formation. How exactly individual histones and histone complexes respond to different repetitive DNA sequence patterns remains an open question. This issue is further complicated by the fact that several additional mechanisms influence histone-DNA binding in promoter regions. Namely, genome-wide, *in vitro* nucleosome reconstruction experiments demonstrate that nucleosome-free regions (NFR) can be formed to some extend even in the mixture of purified genomic DNA with histones [44, 45]. However, intrinsic DNA sequence preferences of nucleosomes still remain an open issue [46]. In particular, it has been recently demonstrated that AT-rich sequences present in many NFRs have little effect on the stability of nucleosomes [46]. Rather it appears that ATP-dependent chromatin modifiers constitute a major factor regulating nucleosome-binding preferences *in vivo* [43, 46].

### *In vitro* protein-DNA binding measurements for ~90 TFs to ~45,000 short, non-genomic DNA sequences validate the nonconsensus binding mechanism

**Fig 4:**
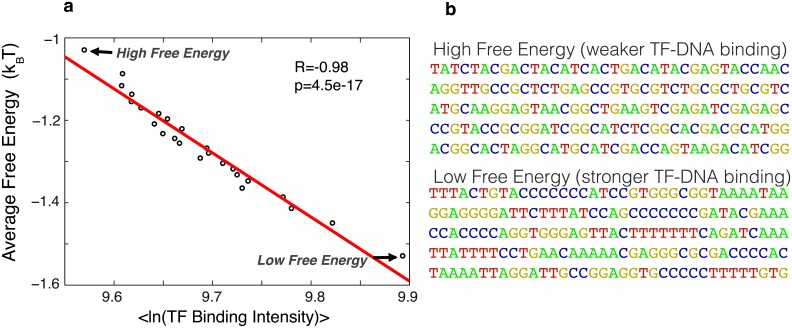
Random-binder model for nonconsensus protein-DNA binding provides a good statistical description of the TF-DNA binding strength measured *in vitro*. **(a)** Plots show the correlation between the free energy of nonconsensus TF-DNA binding, 〈*f*〉, and the measured average TF binding intensity of 82 mouse TFs [34]. We used the data from the PBM experiments performed on universal arrays, which provide measurements of TF binding to all possible 8-bp sequences (8-mers). The data for average TF intensity and free energy was grouped into 25 bins. **(b)** Typical examples of sequences with high and low values of the nonconsensus free energy, respectively, as determined by the *in vitro* PBM measurements.

Here we provide an additional, highly significant validation for the proposed mechanism of nonconsensus protein-DNA binding by the analysis of the available *in vitro* TF-DNA binding data obtained using the protein-binding microarray (PBM) technology [35, 47–49]. The PBM technology allows to simultaneously measure binding of a TF to tens of thousands of 36-bp long DNA sequences in a single experiment [35]. The PBM method is free from the confounding factors, such as the effect of competing TFs and nucleosomes on TF-DNA binding preferences. Here, we used the currently available ‘universal PBM’ data for 91 TFs (belonging to 22 distinct DNA-binding domains) from *C*. *elegans*, *D*. *melanogaster*, and *mus musculus* [33, 34, 50] (Fig 4 and S1 Table). The DNA libraries used in these ‘universal PBM’ experiments were designed in such a way that they cover all possible 8-mer DNA sequences [35], giving an unbiased view of TF-DNA binding specificity. Overall, there are ~45,000 distinct DNA sequences in this library, and thus the TF-DNA binding strength was measured for each TF to all these sequences [33, 34, 50].

We computed the nonconsensus TF-DNA binding free energy, 〈*f*〉_*TF*_, for each 36-bp long DNA sequence in the library using the procedure described above (Fig 1). Contrary to the case of genomic sequences, here we do not move the sliding window along the DNA sequence since each sequence is short, *L* = 36 bp, and therefore a single value of 〈*f*〉_*TF*_ is assigned to each DNA sequence.

Remarkably, for 69 out of 91 analyzed TFs (i.e. 76%) we detected a statistically significant, negative correlation between the nonconsensus protein-DNA binding free energy and the measured *in vitro* TF-DNA binding intensity. This is in agreement with the results obtained for the *in vivo* TF-DNA binding data (Fig 2b and 2d). Twelve TFs (i.e. 13%) did not show a statistically significant correlation, and interestingly, ten TFs (i.e. 11%) showed an *opposite*, positive correlation (S1 Table). The latter observation is remarkable, since it demonstrates that a non-negligible fraction of TFs can respond to DNA symmetries (represented by our free energy model) in an opposite way compared to the majority of other TFs. However, statistically, the *average* TF-DNA binding preferences show highly significant, negative correlation with the computed free energy of nonconsensus protein-DNA binding (Fig 4) in agreement with the *in vivo* results (Fig 2b and 2d).

In order to identify what structural and sequence features are responsible for the anomalous behavior of these 11% of TFs, we classified all TFs according to the DNA-binding domain (DBD) families they belong to. However, we have not identified any particular DBD families that are unique to those 11% of TFs (S1 Table and S4 Fig). We have also not identified any preference of these TFs with respect to any particular biological function, according to the gene ontology (GO) classification. Therefore, the question what sequence and structural features of proteins are responsible for the positive correlation between the free energy and the experimentally measured *in vitro* TF occupancy remains open.

Next, in order to identify protein sequence features that might be responsible for enhanced nonconsensus TF-DNA binding, we separated TFs (we used 82 mouse TFs for this analysis) into two groups. The first group contained 41 TFs with the strongest negative correlation between the free energy and the measured TF occupancy. The second group contained the remaining 41 TFs. We have analyzed the amino acid correlation properties in these two groups of TFs. Our working hypothesis here is that enhanced amino acid sequence correlations in TF sequences are responsible for enhanced nonconsensus TF-DNA binding. We use the term “sequence correlations” in order to describe repetitive sequence patterns. We have previously used a similar analysis in order to investigate protein sequence features responsible for enhanced level of protein structural disorder and protein-protein interaction promiscuity [36]. In particular, we have analyzed the frequency of occurrence of the following repetitive amino acid sequence patterns in each TF group: [*aa*], [*aXa*], [*aXXa*], and [*aXXXa*], where *a* represents each amino acid type and *X* represents an arbitrary amino acid (S2 Table). For example, when we compute the frequency of [Lys-*X*-Lys] pattern, we count the total number of the occurrence of this pattern in each protein sequence, irrespectively to the identity of *X*. As a result of this analysis, we have identified three patterns that demonstrated a statistically significant difference of frequencies between the two TF groups: [Lys-*XX*-Lys] (enriched in the first TF group; Kolmogorov-Smirnov p-value, *p*_*ks*_ ≃ 0.01), [Arg-Arg] (enriched in the second TF group; *p*_*ks*_ ≃ 0.02), and [Leu-*X*-Leu] (enriched in the first TF group;*p*_*ks*_ ≃ 0.05) (S2 Table). In addition the overall compositional fraction of Lys was enriched in the first TF group (*p*_*ks*_ ≃ 0.01) (S2 Table). The fact that the most statistically significant enrichment (distinguishing the two TF groups) is observed for the [Lys-*XX*-Lys] and [Arg-Arg] patterns is encouraging since positively charged Lys and Arg are obviously the key amino acids responsible for TF binding to the negatively charged DNA molecule.

Two conclusions can be drawn from our results. First, that the intrinsic propensity for nonconsensus protein-DNA binding is imprinted both into the DNA and the protein. Since our simple nonconsensus binding model treats proteins as random binders, it captures general trends in the binding profiles of most, but not all, TFs. Second, nonconsensus and specific (consensus) protein-DNA binding mechanisms are tightly interlinked, and both of these mechanisms cooperate in determining the overall protein-DNA binding preferences in eukaryotic genomes. The fact that our simple random-binder model (without any fitting parameters and without any protein-DNA binding specificity built in) provides such a good statistical description of the measured DNA binding strength for the majority of TFs strongly suggests that the nonconsensus mechanism is quite general and it represents the statistical law rather than the exception. However, more accurate, atomistic models describing nonconsensus protein-DNA binding interactions are necessary in order to improve the accuracy of our predictions for different proteins.

## Discussion

Our analyses of the effect of nonconsensus protein-DNA binding demonstrate that the combined genome-wide binding preferences of 69 TFs in *C*. *elegans* and 30 TFs in *D*. *melanogaster* are significantly, negatively correlated with the predicted nonconsensus free energy landscape (Fig 2). Our analyses also show that the experimentally derived nucleosome occupancy in *C*. *elegans* and in *D*. *melanogaster* is significantly, positively correlated with the predicted nonconsensus protein-DNA binding free energy (Fig 3). This trend is qualitatively similar to the one that we previously observed in yeast [12]. The results shown in Figs 2 and 3 strongly suggest that TFs compete with nucleosomes for nonconsensus binding to DNA. Such a competition between TFs and nucleosomes could lead to the enhanced TF binding cooperativity previously predicted by Mirny [51] and Teif et al. [52]. We suggest that nonconsensus protein-DNA binding greatly enhances such nucleosome-induced cooperativity between TFs, and most importantly, in order to achieve this enhancement, promoters do not require the presence of specific, consensus TF binding sites. We stress the important point that the predicted effect of nonconsensus TF-DNA binding most likely affects many but not all TFs. We expect for example, that stress response TFs, such as for example Msn2 in yeast [53], might be insignificantly influenced by the nonconsensus mechanism.

Our model predicts that genomic loci enriched with repetitive sequences, such as in heterochromatin, should also be enriched with TF binding. However, the ChIP-seq analysis in such regions is impeded by the fact that multi-mapping reads from long repetitive region will be filtered out by most peak-calling algorithms, therefore identifying interactions in these regions remains a challenging problem [54]. Interestingly, there are evidences that regions of heterochromatin are not actually transcriptionally inert and non-coding RNA molecules are transcribed from repeated DNA sequences in pericentromeric heterochromatin in different eukaryotic genomes [55]. A recent study even demonstrated [56] that some TFs bind directly to the major satellite repeat DNA sequences that are present in pericentromeric heterochromatin regions and might play a significant role in the mouse heterochromatin formation. Further experiments and analysis of TF binding to the heterochromatin would reveal whether nonconsensus binding play an important role in these regions as well.

Our analysis of available *in vitro* TF-DNA binding data from protein-binding microarray (PBM) experiments (Fig 4 and S1 Table) demonstrates that statistically, on average, *in vitro* TF-DNA binding preferences negatively correlate with the computed nonconsensus free energy landscape, and showed qualitatively similar behavior to the one observed *in vivo* (compare Fig 2b and 2d with Fig 4). This additional analysis is important for several reasons. First, the *in vitro* TF-DNA binding preferences are not affected by the presence of other proteins and histones, which can compete with the protein or cause an indirect binding to the DNA. Second, the TF binding intensity is measured in PBM experiments at significantly higher accuracy compared to ChIP-seq experiments. Third, the usage of non-genomic sequences that cover all possible 8-mer DNA sequences, eliminates possible sequence bias that might exist in the genomic sequences, and thus PBM measurements provide an entirely independent validation of the nonconsensus protein-DNA binding effect. Finally, the present analysis performed for ~90 TFs extends our previous analysis performed for only 6 TFs [15] by more than an order of magnitude, thus strongly suggesting the generality of the nonconsensus protein-DNA binding effect in eukaryotic genomes.

Interestingly, ten TFs (i.e. 11%) showed an *opposite*, positive correlation between the free energy and the measured TF-DNA occupancy (S1 Table). The latter observation is remarkable, since it demonstrates that a non-negligible fraction of TFs can respond to DNA symmetries (represented by our free energy model) in an opposite way compared to the majority of other TFs. However, we failed to identify any particular structural, sequence, or functional features unique to this set of TFs. This failure might stem from the small number of proteins that exhibited such behavior. Yet, we were able to identify repetitive amino acid sequence patterns that are responsible for enhanced nonconsensus TF-DNA binding (S2 Table). In particular, for the group of TFs characterized by the strongest nonconsensus TF-DNA binding preferences, the most statistically significant enrichment is observed for the [Lys-*XX*-Lys] pattern, while the frequency of [Arg-Arg] pattern is reduced in this group (S2 Table). The latter result is intuitively sound since both Lys and Arg are the key amino acids responsible for TF binding to the negatively charged DNA molecule.

Importantly, in this study, our random-binder statistical mechanics model for protein-DNA interactions does not use any experimentally pre-determined information on either low-affinity or high-affinity TF-DNA binding sites. The genomic DNA sequence constitutes the only experimental parameter of the model. In addition, our model does not have any fitting parameters. Contrary to the case of specific protein-DNA binding that requires the presence of a 6 to 20-bp long specific DNA motif (unique for each individual TF), the nonconsensus protein-DNA binding effect stems from multiple nonspecific interactions between the TF and a relatively long (few tens of bp) DNA fragments enriched with repetitive sequence patterns. The fact that different TFs are affected in a *statistically similar* way by *entirely different DNA sequences* containing similar repetitive patterns constitutes the key difference between the nonconsensus and specific protein-DNA recognition modes.

What exactly is the interplay between nonconsensus DNA repetitive sequence elements and consensus (specific) sequences and how their combination influences the overall binding of proteins to the DNA and the expression levels of genes are important questions yet to be explored. We suggest that repetitive nonconsensus sequence elements might have similar influence on TF-DNA binding and on gene expression as repeats of consensus (specific) DNA sequence elements (i.e. homotypic clusters) [57]. However, an important difference between these two types of repeated sequence elements is that nonconsensus repeats can affect many different TFs in a similar way, while homotypic clusters are more specific to a limited set of TFs.

Repetitive sequence elements located near the consensus (specific) motif, could increase the TF association rate, by inducing the one-dimension “sliding” of the TF, and improving its search for the specific binding site [20, 58]. The presence of many weaker sites flanking a strong binding site could lead to a funnel effect [59–62], where the molecules are directed to the strong binding site as depicted in S5a Fig. It could also stabilize binding sites that are not strong enough individually [63, 64] and increase the ability of binding sites to “withstand mutations” [65]. We use the *C*. *elegans* Hlh-1 protein as an example demonstrating that nonconsensus DNA sequence elements might stabilize the binding to specific consensus elements *in vivo* (S5b Fig). The analysis of Hlh-1 binding sites (based on the genome-wide ChIP-seq measurements [2, 3] in *C*. *elegans*) demonstrates that only 5% of the total number of Hlh-1 specific motifs in the genome is bound by Hlh-1 (S5b Fig). We sorted the genomic sequences containing the Hlh-1 motif (consensus motifs were reported in [3]) into two groups: the first group contains DNA sequences that were experimentally determined as being bound by Hlh-1, while the second group contains unbound DNA sequences. S5c Fig represents the average nonconsensus protein-DNA binding free energy computed for each of these two sequence groups. We observed that the nonconsensus free energy is reduced for the group that contains bound sequences as compared with the group that contains unbound sequences. The computed *p*-values show that this result is statistically significant (S5c Fig). This example supports the hypothesis that nonconsensus sequence elements might provide the funnel effect *in vivo*. Additional analysis and experimental measurements of the kinetics of TF-DNA binding to consensus (specific) sequence elements embedded in different nonconsensus DNA backgrounds, should shed more light on this hypothesis.

Future *in vitro* measurements of binding preferences for additional TFs [66], combined with high-resolution *in vivo* ChIP-seq and ChIP-exo analysis, will help to complete the molecular picture of design principles for nonconsensus protein-DNA binding and its functional significance.

## Methods

### Gene sets

We used the set of 23,287 *C*. *elegans* genes based on Wormbase annotation, WS228 [2, 67], and 12,188 *D*. *melanogaster* genes annotated in [10].

### Experimental *in vivo* TF occupancy

We used experimentally measured binding preferences of 69 *C*. *elegans* TFs (S3 Table), as determined by the Gerstein and Snyder labs [2, 3]; for computing the *D*. *melanogaster* TF occupancy we used binding preferences of 30 TFs (S4 Table) determined by the White lab [8]. TF-DNA binding preferences for both genomes were measured using ChIP-seq assays (modENCODE project). We defined TF occupancy for each genomic location as the total number of bound TFs at each location along the genome.

### Experimental nucleosome occupancy

We used experimentally measured, genome-wide, normalized nucleosome occupancy determined by the paired-end Ilumina sequencing in *C*. *elegans* [4, 5]; we also used the genome-wide map of H2A.Z nucleosome occupancy in *D*. *melanogaster* embryos (0–12 hr) (determined in [32]).

### Experimental *in vitro* TF-DNA binding strength measured using PBM

We used experimentally measured *in vitro* binding intensity for the *C*. *elegans*, *D*. *melanogaster*, and *mus musculus* TFs (S1 Table), determined using the protein-binding microarray (PBM) technology [33, 35, 47–49].

### Calculation of the free energy of nonconsensus protein-DNA binding

In order to compute the nonconsensus protein-DNA binding free energy landscape, we generate an ensemble of random DNA binders as a proxy for the phenomenon of nonconsensus protein-DNA binding in a crowded cellular environment [11]. Our model does not use any experimentally pre-determined protein-DNA binding preferences in order to model protein-DNA binding. The actual DNA sequences of the *C*. *elegans* and *D*. *melanogaster* genomes constitute the only input parameter for our model. In order to compute the free energy of nonconsensus protein-DNA binding at any given location along a DNA sequence, we position the center of the sliding window of width L = 50 bp at that location. The 50 bp length is a typical sliding event distance of a protein along the DNA under physiological conditions [68, 69] (Fig 1).

We assume that a model protein (random binder) makes *M* bp contacts with the DNA (Fig 1b) and that the model protein-DNA interaction energy at each genomic position *i* is simply a sum of *M* interaction energies:

$$U(i)=-\sum_{j=i}^{M+i-1}\sum_{\alpha =\{A,T,C,G\}}K_{\alpha }s_{\alpha }(j)$$ (1)

where *s*_*α*_(*j*) represents the elements of a four-component vector of the type (*δ*_*αA*_, *δ*_*αT*,_ *δ*_*αC*,_ *δ*_*αG*_), and *δ*_*αβ*_ = 1 if *α* = *β*, or *δ*_*αβ*_ = 0 if *α* ≠ *β*. For example, if the A nucleotide is positioned at the coordinate j along the DNA, then this vector takes the form: (1,0,0,0). If, for example, the DNA sequence contains entirely poly(A) at a given genomic location, then a random binder makes all *M* contacts with the A nucleotide, and hence at this location the resulting energy, Eq (1), will be simply, *MK*_A_. In order to generate each model protein, we draw the values of *K*_*A*_, *K*_*T*_, *K*_*C*_, and *K*_*G*_ from Gaussian probability distributions, *P*(*K*_*α*_), with zero mean, and standard deviation *σ*_*α*_ = 2*k*_*B*_ *T*, where *T* is the temperature and *k*_*B*_ is the Boltzmann constant. We have shown previously that the resulting free energy is qualitatively robust with respect to the choice of model parameters [11]. The energy scale, 2*k*_*B*_ *T* ≃ 1.2 kcal/mol, is chosen to represent a typical strength of a hydrogen bond, or an electrostatic bond that a protein makes with one DNA bp [16, 19].

For each model random binder, we define the partition function of protein-DNA binding within the chosen sliding window of width *L* bp:

$$Z=\sum_{i=1}^{L}\text{exp}(-U(i)/k_{B}T)$$ (2)

and the corresponding free energy of *nonconsensus* protein-DNA binding in this sliding window:

$$F=-k_{B}T\text{ln}Z$$ (3)

We then assign the computed *F* to the sequence coordinate in the middle of the sliding window. Next, we move the sliding window along the DNA sequence and we compute *F* at each sequence location. This procedure allows us to assign the *free energy of nonconsensus protein-DNA* binding to each DNA bp within the genome.

Next, we repeat the described procedure for an ensemble of 250 model random binders (Fig 1) and compute the average free energy, 〈*F*_*TF*_〉, over this ensemble, at each sequence location. We stress that the resulting free energy is qualitatively robust with respect to the choice of the sliding window size, *L*, within a wide range of values (S1 Fig). In addition, the free energy profiles are statistically robust with respect to a moderate variation of the value of *M*, within a typical range of the TF binding site size (S1 Fig). We verified that the predicted free energy landscape is dominated by DNA sequence correlations, and *not* by the average nucleotide composition (S2 Fig). In particular, for each random binder, in each sliding window we computed the normalized free energy, *δF* = *F*−*F*_*rand*_, where *F*_*rand*_ is the free energy computed for a randomized sequence (in the same sliding window as *F*) and averaged over 25 random realizations.

### *p*-value calculations

In order to compute the *p*-value for S5c Fig, we first selected all the 800 bp-long sequences containing the exact binding motifs for each TF. For example, genome-wide, we have overall 9258 sequences containing the consensus Hlh-1motif. Among those 9258 sequences, 442 sequences were experimentally determined as bound by Hlh-1, while the rest of 8816 sequences were unbound. In order to compute the *p*-value, we compiled 10^5^ pairs of groups containing 442 and 8816 sequences, respectively, randomly chosen from the original 9258 sequences. These 10^5^ pairs of groups represent randomized analogs for the original groups of bound and unbound Hlh-1motifs. Second, for each of these pairs of random groups we computed the average free energies, 〈*f*〉, of nonconsensus binding separately for the randomized bound and unbound groups, as described above. Third, for each pair of randomized groups we computed the difference of the integrated free energy within the interval (-400,400) between the two randomized groups. Finally, we computed the probability that this difference is equal or larger than the actual value of the difference. The latter probability was taken as the *p*-value.

## Supporting Information

S1 FigRobustness of the computed free energy of nonconsensus protein-DNA binding with respect to (A) the TFBS size, *M*, and (B) the width of the sliding window, *L*.Plots show the normalized, average free energy per bp, $\langle \delta f\rangle ={\langle {\langle \delta F\rangle }_{TF}\rangle }_{seq}/M$, where *δF* is computed in the interval (-400,400) around the TSSs of 18,150 genes in *C*. *elegans*. The free energy *F* is computed as described in the main text, using an ensemble of 125 random DNA binders. *F*_*rand*_ is the free energy computed for a randomized sequence (in the same sliding window as *F*), and averaged over 25 random realizations.(EPS)

S2 FigRobustness of the computed free energy of nonconsensus protein-DNA binding with respect to the global variability of the nucleotide content along the genome.Plot shows the average free energy per bp, 〈*f*〉 (blue curve) compared to the corresponding *normalized* average free energy $\langle \delta f\rangle ={\langle {\langle \delta F\rangle }_{TF}\rangle }_{seq}/M$ (red curve), where *δF* = *F*−*F*_*rand*_. Both the un-normalized and normalized energies are plotted in the interval (-400,400) around the TSSs of 18,150 genes in *C*. *elegans*. The free energy *F* is computed as described in the main text, using an ensemble of 125 random DNA binders. *F*_*rand*_ is the free energy computed for a randomized sequence (in the same sliding window as *F*), and averaged over 25 random realizations. We used *M = 8* and *L = 50* in our calculations. The described procedure removes a possible bias in the free energy stemming from the global variability of the nucleotide content.(EPS)

S3 Fig Illustration of the entropy-dominated mechanism for nonconsensus TF binding to repetitive DNA sequence elements. **(a)** Probability distribution, *P*(*U*), for two different groups of DNA sequences: the first group is composed of 1000 genomic (*C*. *elegans*) DNA sequences containing repetitive elements (black), and the second group of sequences is composed of randomly permuted DNA sequences from the first group (gray). The repetitive, genomic sequences are characterized by a wider standard deviation of *P*(*U*) than the randomly permuted, non-repetitive sequences. The length of each sequence is 58-bp; the sequences were selected from the TSS region of the *C*. *elegans* genes. (**b**) The repetitive DNA sequences are characterized by the lower (statistically, on average) free energy of nonconsensus TF-DNA binding, <*F>*_*TF*_ than the randomly permuted, non-repetitive DNA sequences.
(EPS)

S4 Fig Classification of TFs with respect to their DNA-binding domain (DBD) families and the correlation between TF occupancy and free energy of nonconsensus TF-DNA binding. Each box-plot represents **(a)** DBD family, where each TF belonging to this family is represented by a single bar. The *y*-axis displays the correlation *R*-value between the measured *in vitro* TF occupancy and the free energy of nonconsensus DNA-binding. Most of the DBD families only contain TFs with negative *R*-values. One family contains two proteins with positives *R*-values (SAND). Three DBD families contain TFs that are both negatively and positively correlated with the free energy (BRLZ, GATA, HLH). Fig shows only the DBD families that contain at least two TFs (15 out of 22 DBD families), and only mouse TFs (74 out of 91 PBM-tested TFs).
(EPS)

S5 FigExample of how nonconsensus sequence elements can influence consensus (specific) TF-DNA binding to the specific TFBS.
**(a)** Schematic representation of the nonconsensus funnel effect. Repetitive, nonconsensus sequence elements can increase the TF binding to the DNA near a strong, specific binding site, and to induce a one-dimension “sliding” of the TF towards the specific TFBS. **(b)** Two examples of sequences, both containing exactly the same consensus-binding motif ACAGCTG for the *C*. *elegans* transcription factor Hlh-1, surrounded by different nonconsensus sequence elements (Hlh-1was detected as bound [2, 3] to the specific binding motif shown in the top sequence, but it remained unbound to the *identical* specific motif shown the bottom sequence). **(c)** The computed average free energy per bp, $\langle \delta f\rangle ={\langle {\langle \delta F\rangle }_{TF}\rangle }_{seq}/M$, in the interval (-400,400) around Hlh-1specific motifs that was detected as being bound (red). Blue line corresponds to 〈*f*〉 computed for DNA sequences surrounding unbound Hlh-1motifs [2, 3]. The specific motifs, which were detected as being bound, are surrounded by DNA sequences with significantly lower average free energy compared to unbound motifs (computed *p*-value < 10^−5^; see Methods).(EPS)

S1 Table The table shows the correlation between the computed free energy of nonconsensus TF-DNA binding, *f*, and the measured TF binding intensity for 91 TFs from [33, 34,47,50]. The linear correlation coefficient, *R*, and the *p*-value were calculated for each TF after binning the data into 50 bins (the binning is performed in a way similar to the binning performed in the plots presented in the main text). 69 out of 91 proteins statistically behave according to our model (*p* < 0.05), ten proteins exhibit the opposite behavior (*p* < 0.05), while the remaining 12 proteins show no statistically significant correlation (*p* > 0.05). For each TF in the table, the protein name and its DNA-binding domain type are specified.
(XLSX)

S2 Table Statistical analysis of repetitive amino acid patterns and the amino acid content of TF sequences belonging to the two groups of the mouse TFs (82 TFs overall). The first group (1st average) contained 41 TFs with the strongest negative correlation between the free energy and the measured TF occupancy. The second group (2nd average) contained the remaining 41 TFs. We have analyzed the frequency of occurrence of the following repetitive amino acid sequence patterns in each TF group: [*aa*], [*aXa*], [*aXXa*], and [*aXXXa*], where *a* represents each amino acid type and *X* represents an arbitrary amino acid. The second table contains the average amino acid content in each group of TFs. The presented p-values represent the Kolmogorov-Smirnov p-values.
(XLSX)

S3 Table List of modEncode *C*. *elegans* TFs. (XLSX)

S4 Table List of modEncode *D*. *melanogaster* TFs. (XLSX)
